# The Influence of Nitrogen Application Level on Eating Quality of the Two Indica-Japonica Hybrid Rice Cultivars

**DOI:** 10.3390/plants9121663

**Published:** 2020-11-27

**Authors:** Xincheng Zhang, Liangbo Fu, Yishan Tu, Huifang Zhao, Liuhui Kuang, Guoping Zhang

**Affiliations:** Department of Agronomy, Key Laboratory of Crop Germplasm Resource of Zhejiang Province, Zhejiang University, Hangzhou 310058, China; 0617375@zju.edu.cn (X.Z.); fulb@zju.edu.cn (L.F.); 21916141@zju.edu.cn (Y.T.); 11716028@zju.edu.cn (H.Z.); kuangliuhui@zju.edu.cn (L.K.)

**Keywords:** indica-japonica hybrid rice, eating quality, nitrogen, inferior grains, protein fractions

## Abstract

Indica-japonica hybrid rice cultivars show great yield potential but poor eating quality and require more nitrogen (N) input relative to japonica rice. However, the effect of N levels on the eating quality of indica-japonica hybrid rice is little known. A field experiment was carried out to investigate the effects of four N levels on two indica-japonica hybrid rice cultivars (Yongyou12 and Yongyou17) differing in eating quality. The results showed that the contents of amylose chains and water-insoluble storage proteins, especially prolamin, increased largely under a high N level, leading to deterioration of the rice-eating quality, although a low N level (100 N kg/ha) had a less negative effect on the eating quality. Moreover, both of the indica-japonica hybrids had high ratios of inferior grains (IG), and the ratio of IG increased with the N level. Grain weight and the immature ratio of IG were reduced and increased with the N level, respectively, which are also factors for deterioration of the eating quality. The two cultivars differed greatly in the responses of eating quality to the N level, with Yongyou17 being more sensitive than Yongyou12. The current results indicated that a high N level deteriorates the eating quality of indica-japonica hybrid rice mainly due to a large increase of IG.

## 1. Introduction

Rice is a major cereal crop that provides food for nearly half of the world’s population. With the rapid improvement of living standards, people pay more attention to the eating and cooking quality of rice [1]. In recent years, indica-japonica hybrid rice cultivars were widely released and planted in Southern China, which is characterized by high grain yield and relatively poor eating quality [2,3]. The eating quality of rice mainly includes the texture and flavor of cooked rice [4] and could be directly or indirectly reflected by taste value, breakdown (BD) and setback (SB) [1,3]. Recently, Bian et al. reported that the protein content was a major factor affecting the rice-eating quality in a study using two indica-japonica hybrid cultivars differing in panicle types and Satake Rice Taste Analyzer (STA) [3].

The eating quality of rice is mainly controlled by genetics [1] and, also, affected by environmental factors, including CO_2_ concentration [5], temperature [6] and nitrogen rate [7,8,9], and so on. Nitrogen is an important nutrient affecting rice yield and quality. The effects of nitrogen fertilizer on the eating quality of rice was intensively investigated, but so far, the results are inconsistent and controversial. It is commonly considered that more N fertilizer application commonly causes a deterioration of the eating quality [7,8,9,10]. Cao et al. found that, under a high N level, the apparent amylose content (AAC) and setback (SB) increased, while the gel consistency (GC), breakdown (BD) and peak viscosity (PV) decreased, thus deteriorating the eating quality [9]. Yang et al. reported that a high N level reduced the short-chain proportion of amylopectin, leading to a poor eating quality [8]. However, Duan et al. found opposite results [11]. The application of N fertilizer at the heading stage caused a declined rate of starch accumulation at the grain-filling stage under high temperature conditions, which mitigated the deterioration of the rice quality induced by high temperatures [12]. N fertilizer application at a proper rate may increase the micronutrient and protein contents in grains, thus improving the nutrition quality of rice [13]. In comparison with conventional rice, indica-japonica hybrid rice requires much more N fertilizer for their normal growth and yield. However, the effect of N levels on the eating quality of indica-japonica hybrids remains little known. Within a rice panicle, florets can be divided into superior and inferior ones according to their differentiating times and locations on a panicle and rachis. Superior grains (SG) develop earlier and are better-filled, thus being larger and heavier than inferior grains (IG) [14]. Therefore, SG and IG differ in their chemical composition and eating quality [15,16,17]. In general, superior grains have higher amylose contents but lower protein contents than inferior grains [15]. However, the opposite results were found by Ma et al. [16]. The asynchronous filling among florets in a panicle is more serious for indica-japonica hybrid cultivars than conventional rice, probably due to the larger panicle with much more florets, which could be partially attributed to the relatively poor eating quality of the hybrid rice.

Is a high N level a major factor for deteriorating the eating quality of the hybrid rice? Or is the difference in asynchronous filling between SG and IG enlarged under a high N level, which, in turn, deteriorates the rice-eating quality? Accordingly, the current experiment was performed to study the influence of four N fertilizer levels on the eating quality of two indica-japonica hybrid rice cultivars differing in eating quality through determining Rapid Visco Analyser (RVA) profiles, contents of amylose, protein and its fractions and starch structure, as well as the taste parameters of cooked rice.

## 2. Results

### 2.1. Effects of N Treatments on the Eating Quality

**Figure 1:**
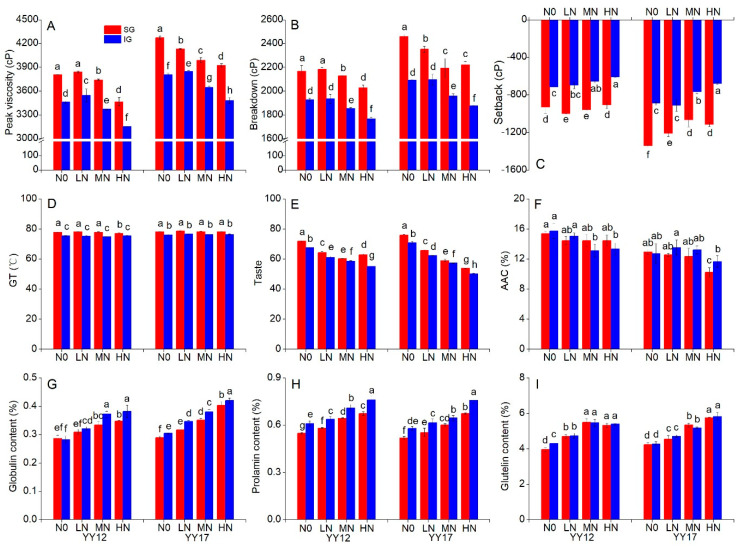
The peak viscosity (**A**), breakdown (**B**), setback (**C**), gelatinization temperature (**D**), taste (**E**), apparent amylose content (**F**), globulin content (**G**), prolamin content (**H**) and glutelin content (**I**) of the superior and inferior grains under different nitrogen levels. YY12, Yongyou12; YY17, Yongyou17; SG, superior grains; IG, inferior grains; GT, gelatinization temperature; AAC, apparent amylose content; N0, control; LN, low nitrogen; MN, medium nitrogen and HN, high nitrogen. Different letters labeled on the columns in the same cultivar are significantly different (*p* < 0.05).

The RVA profile is generally used as an indirect indicator for the evaluation of the rice-eating quality. The pasting parameters of rice flour differed significantly among the four nitrogen levels. There was no significant difference in PV, BD and SB between low nitrogen (LN) and the control (N0), while PV and BD were significantly reduced, and SB was significantly increased in both medium nitrogen (MN) and high nitrogen (HN) treatments for the two cultivars (Table 1). In the SG sample, the PV value showed the same trend with that in all grains (AG) in responding to the N level, while it was significantly increased in the IG sample under LN (Figure 1A). BD and SB in the SG and IG samples did not significantly differ from those in AG in responding to the N level (Figure 1B,C). In comparison with the control, a high N treatment caused a marked deterioration of the rice-eating quality. On average of the four N treatments, the PV and BD values were significantly higher in YY17 than in YY12, while the SB value was just the opposite for the two cultivars (Table 1). Obviously, YY17 is better in the eating quality than YY12. In addition, IG showed much lower PV and BD but higher SB than SG. It is noted that PV and BD in the SG sample from HN were still much higher than those of IG from N0; the opposite trend was observed for SB (Figure 1A–C). There was no significant difference in gelatinization temperature (GT) among the four nitrogen treatments for both SG and IG, while the value was markedly lower in IG than in SG for the two cultivars (Figure 1D).

The taste measurements of the cooked rice can provide information about the palatability. With increased N levels, the rice appearance, viscosity, balance and taste values were remarkably reduced, while hardness was increased for the two cultivars (Table 1). The difference in the taste parameters was much smaller between the N0 and LN treatments than between the LN and HN treatments, indicating rice palatability is easily affected by a high N fertilizer. In addition, YY17 showed a greater change than YY12 in the taste value from N0 to HN (Table 1). The same trend in responding to the N level was found in SG and IG, and SG showed a better palatability than IG in any treatment (Figure 1E and Figure S1C–E). On the other hand, the gel consistency showed the opposite trend with hardness in responding to the N level (Table 1). Thus, it can be seen from the taste parameters and GC that YY17 keeps a better palatability than YY12 in any treatment, which supported the results of the RVA analysis. 

### 2.2. Effects of N Treatments on Endosperm Compositions

The nitrogen level had substantive effects on the endosperm components related to the eating quality (Table 2). The AAC was reduced with the increased N levels for the two rice cultivars. A similar trend was also found in both SG and IG (Figure 1F). On average of all the N levels, the AAC of YY17 (12.20%) was slightly lower than that of YY12 (13.34%). Among the four protein components, the albumin content had little change; the globulin, prolamin and glutelin, as well as total protein contents, showed significant increases with the increased N levels for the two cultivars (Table 2). No significant difference was found in all protein fractions between the two cultivars in any N level, except prolamin, which was higher in YY12 than YY17 (Table 2). The SG and IG samples showed similar trends (Figure 1G–I). However, there was no significant change in the pro/pro+glu ratio among all N treatments. The prolamin content and the ratio of prolamin to prolamin+glutelin (pro/pro+glu ratio) were significantly higher in YY12 than in YY17 at any N level (Table 2). Interestingly, the globulin and prolamin contents of IG were markedly higher than those of SG at any N level, while there was no significant difference in glutelin between the SG and IG samples (Figure 1G–I).

**Figure 2:**
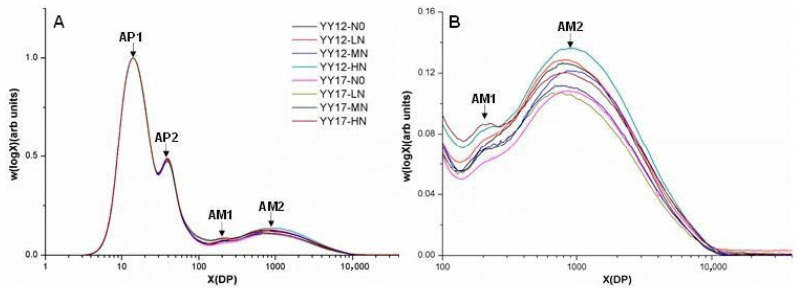
(**A**) Size exclusion chromatography (SEC) weight chain length distribution (CLDs), w(log X), of the debranched starches and (**B**) an enlargement of the amylose region as a function of the degree of polymerization (DP) X. AP1 and AP2 stand for the two amylopectin peaks, while AM1 and AM2 stand for the two amylose peaks.

The starch structure parameters of all samples were shown in Table 3 and Figure 2. As the degree of polymerization (DP) at peak AM1 (X_AM1_) and the ratio of peak AM1 height to peak AP1 height (h_AM1/AP1_) of YY12 in N0 was not detected, these two parameters were not used for the comparisons among treatments and between the two cultivars. The nitrogen level had little influence on the DP at four peaks, i.e., AP1, AP2, AM1 and AM2 (X_AP1_, X_AP2_, X_AM1_ and X_AM2_), respectively, but affected the h_AM1/AP1_, the ratio of peak AM2 height to peak AP1 height (h_AM2/AP1_) and amylose contents (AC). The ratio of peak AP2 height to peak AP1 height (h_AP2/AP1_) was remarkably higher in YY17 than in YY12, while the long amylose chains (h_AM2/AP1_) was just the opposite for the two cultivars.

### 2.3. The Influence of N Level on the Grain Appearance of SG and IG

The grain shape of brown rice varied with their spike positions (Table 4 and Figure S2). On average of all the N levels, the ratio of IG to total grains per spike was two times more than that of SG. The ratios of SG were decreased with the application of nitrogen, while that of IG showed the opposite trend. Compared with N0, the grain length and width of SG and IG were significantly decreased in HN, while remained slightly changed in the other N treatments. The grain length/width of both SG and IG showed substantial differences for YY12 and little change for YY17 among the four N treatments (Table 4). The grain weights of both SG and IG were significantly reduced under high N conditions. Between the two cultivars, YY17 had a larger grain length and length/width ratio but smaller grain width than YY12. The grain length, width, thickness and weight of SG were significantly higher than those of IG at any nitrogen level.

With increased N levels, the green rice kernel (GRK) and died rice kernel (DRK) ratios of IG were significantly increased, while those of SG were not detected or very small to be ignored (< 1%). For IG, in GRK occurrence, YY17 was more sensitive to N than YY12 (Table 4 and Figure S2). In addition, the NRK ratio declined dramatically with increased nitrogen levels.

## 3. Discussion

### 3.1. High N Level Deteriorates the Eating Quality of Rice

The high eating quality of rice is generally characterized by a high PV and BD, low SB, high balance, taste value, GC and low hardness [7,17,18]. A number of researchers have studied the effects of the nitrogen level on the rice-eating quality, but the results are inconsistent—even controversial—probably due to the difference in rice genotypes, growth conditions and N levels used in their experiments. Gu et al. reported that, with the increased N level, the RVA viscosity decreased and SB increased, resulting in deteriorated rice palatability [7]. Cao et al. found that BD and SB were more sensitive to low nitrogen than to high nitrogen [9]. However, Zhu et al. reported that a nitrogen rate in the range of 0-337.5 N kg/ha had no significant influence on the HPV, CPV and SB values [19]. In this study, there were no significant differences in PV, BD and SB between LN and N0, while these values were dramatically declined in MN and HN. Additionally, the rice palatability was more sensitive to HN than to LN (Table 1). It may be suggested that N fertilizer should be controlled for improving the eating quality of rice. The current results confirmed that excessive N fertilizer application deteriorates the eating quality of the two hybrid cultivars. Moreover, in this study, we found that the eating quality of indica-japonica hybrid rice remained little changed in the range of 0–100 N kg/ha and become worse in the range of 100–200 N kg/ha, indicating it is possible to coordinate the contradiction between the high yield and high quality by controlling the N application.

### 3.2. The Deterioration of Rice-Eating Quality under a High N Level Is Attributed to an Increased Protein Content and Ratios of Amylose Chains

It is well-known that the eating quality of rice is related to its chemical composition—in particular, to the apparent amylose content (AAC). Generally, the higher the AAC, the firmer the starch gel [1], and the lower the BD, the higher the SB [20]. The current results showed that the N fertilizer had a small effect on the AAC, which is beneficial for coordinating the relationship of high yield and high quality. In addition to the amylose content, the starch structure is another important factor affecting the eating quality. Li et al. found that the starch with a similar amylose content but more DP1000-2000 chains would make cooked rice harder [21]. Similar results were obtained in this study, that the samples containing a high ratio of amylose chains (AC, h_AM1/AP1_ and h_AM2/AP1_) showed a harder texture of cooked rice. Moreover, the examined samples with the highest ratio of amylose chains under a high N treatment showed the highest SB and the lowest BD and taste values (Table 1 and Table 2). Thus, it may be concluded that a high N application increased the amylose chains, resulting in deterioration of the eating quality.

It is commonly recognized that the deterioration of the rice-eating quality under a high N level is mainly due to the increased protein content [7,10]. Martin and Fitzgerald confirmed that protein could lower the RVA profile [22]. Furthermore, Baxter et al. demonstrated that water-insoluble storage proteins made a reduction in most RVA viscosities, while water-soluble albumin had the opposite effect [23,24]. In this study, we did not take albumin into consideration, because it was little affected by the N level. With increased N levels, the water-insoluble proteins increased, resulting in a reduction of viscosity (Table 1 and Table 2), which was in line with the previous studies [24,25,26]. It was recently reported that the protein content is a major factor affecting the cooking and eating quality of indica-japonica hybrid cultivars [3]. In this study, we found that YY12 contained a higher prolamin content than YY17, and there was no difference in the other protein fractions between the two cultivars. Therefore, it may be assumed that prolamin may play a more important role than the other protein fractions in determining the rice-eating quality.

### 3.3. Inferior Grains Become Worse in the Eating Quality under a High N Level

In this study, we found that the ratio of IG was almost two times as large as that of SG for the two hybrid cultivars and increased with the application of the nitrogen fertilizer, especially in the HN treatment (Table 4). Moreover, the PV and BD of SG in the HN treatment were still much higher than those of IG in the N0 treatment, and the opposite was true for SB (Figure 1A–C), indicating that IG contributes more to the eating quality than SG. The difference in eating quality between superior and inferior grains results from their different biochemical compositions [16,17]. In this study, IG contained higher globulin and prolamin contents than SG (Figure 1G,H). Meanwhile, IG had lower PV and BD and higher SB than SG (Figure 1A–C). Therefore, the poor eating quality of inferior grains can be described to higher protein, as well as globulin and prolamin, contents. In general, SG have a greater rate of grain filling than IG, resulting in larger grains [27]. In this study, a high N level caused the dramatic reduction in grain length, width, thickness and weight of IG (Table 4). On the other hand, the GRK ratio of IG was dramatically increased under high N levels, which agreed with the previous findings that a high N increased the GRK, leading to poor eating quality [28]. The current results indicate it is important to reduce the ratio of IG and enhance the filling of IG for improving the rice-eating quality.

## 4. Materials and Methods

### 4.1. Materials and Experimental Design

Two indica-japonica hybrid rice cultivars, Yongyou12 (YY12) and Yongyou17 (YY17), were used in this study. A field experiment was conducted at Changxing Experimental Station of Zhejiang University (30°53′37″N, 119°38′13″ E), China in 2018. The soil type was clay, containing 1.05-g/kg total N, 20.43-mg/kg available P and 102.5-mg/kg exchangeable K. The experiment was arranged in a split plot design, with the nitrogen fertilizer treatment as the main plot and variety as the subplot, three replicates for each treatment, and the area of the subplot was 16 m^2^. Each plot was separated by ridges mulched with a plastic film to protect it from leaching. The nitrogen treatment consisted of 4 levels, i.e., 0 (control, N0), 100 (low N, LN), 200 (medium N, MN) and 300 N kg/ha (high N, HN). A nitrogen fertilizer in the form of urea was equally applied at the four growth stages, i.e., before transplanting, tilling stage, panicle initiation stage and booting stage, respectively. In addition, the P fertilizer of 140 kg ha^−1^ in the form of calcium superphosphate and K fertilizer of 186 kg ha^−1^ in the form of potassium chloride were applied before transplanting. Seeds were sown in seedbeds on May 24th, and two seedlings per hill were transplanted to a paddy field on June 24th.

### 4.2. Sampling

At maturity, when husks of all grains in a panicle become golden-yellow, about 200 uniform panicles per treatment were harvested with scissors, and grains were threshed manually. Then, sample grains were dried naturally (around 13% of the moisture content) in a cold chamber at 4 °C for further analysis. The samples from the whole panicle were named as all grains (AG), while other samples were named as superior grains (SG) from the top primary and middle primary branches and inferior grains (IG) from the middle and bottom secondary branches, according to Zhang et al. [29]. The ratios of SG and IG to total grains per panicle were calculated. The sampled grains were dehulled manually for measuring the grain appearance and milled for 1 min by a LTJM-160 rice polisher (OUMiYA, Taizhou, China), and some milled rice was ground into powder.

### 4.3. Grain Shape and Appearance Observation

Ten intact brown rice kernels were selected randomly and placed into a line along the length of the rice grains, and the length was measured by a ruler. Then, the arrangement was remade along the width of the rice grains to determine the rice width. There were three replicates for each measurement. The thickness of the rice grain was measured with a Vernier caliper (Guanglu, 111N-101, Guilin) using 20 grains. Grain weight was determined by weighing 500 brown rice with three replicates.

Three hundred brown rice were categorized into three groups according to Qiao et al., with some modifications [28]: normal rice kernel (NRK, including perfect rice kernel and chalky rice kernel), green rice kernel (GRK) and died rice kernel (DRK, collapsed grains with floury endosperm) (Figure S2).

### 4.4. Taste Measurement of Cooked Rice

Taste of the milled rice was determined using a Satake Rice Taste Analyzer (STA1A, Satake, Japan) to obtain the taste parameters, including hardness, balance and taste. The measuring processes were as follows: 30-g milled rice was put into a stainless-steel tank, washed three times with deionized water, then drained, and water was added to make the ratio of rice to water into 1:1.3. The samples were soaked for 30 min, then heated for 25 min and kept warm for 10 min prior to determination. Finally, 7.0 g of steamed rice was loaded into a stainless-steel ring with a diameter 30 mm and height 9 mm, made into a rice cake and put into the test groove of the taste analyzer, and all readings were recorded.

### 4.5. Measurement of RVA

The pasting properties of milled rice flour were analyzed using a Rapid Visco Analyzer (RVA, model 3D, Newport Scientific, Warriewood, Australia) according to Zhu et al., with small modifications [20]. Briefly, 3-g flour was mixed with 25-mL deionized water in the RVA canister, heated to 95 °C at a rate of 12 °C/min, held at 95 °C for 2.5 min, cooled down to 50 °C at 12 °C /min and, finally, held at 50 °C for 2 min.

### 4.6. Starch Extraction and Measurement

Starch of milled rice was extracted, purified and debranched according to Li et al. [21] The molecular size distribution of debranched starch was determined in duplicate using GRAM 100 and GRAM 1000 columns (PSS, Shanghai) in a column at 80 °C, with the pullulan standards for calibration to obtain a relationship between the size exclusion chromatography (SEC) elution volume and hydrodynamic volume (Vh) of the starch molecules, and the degree of polymerization (DP) of the linear branches was calculated using the Mark-Houwink-Sakurada equation [30]. For debranched starch chains, the relationship between DP and molar mass (M) was determined using the equation: M = 162.2DP + 18.0 (162.2 was the molecular weight of the anhydroglucose monomeric unit, and 18.0 was that of the additional water in the end group).

All SEC weight distributions were normalized to the height of the highest peak (AP1) for a better comparison of the chain length distribution. In order to compare the starch structure quantitatively, a set of structure parameters was defined and obtained from the chain length distribution (CLDs) of the SEC weight. They were the DP at each maximum peak, named as X_AP1_, X_AP2_, X_AM1_ and X_AM2_, according to Wang et al. [31], which reflected the relative sizes of the chains in each group (Figure 2). The height ratio of each maximum relative to that of AP1, h_AP2/AP1_, h_AM1/AP1_ and h_AM1/AP1_ represented the relative amount of chains in each group. The amylose content (AC) was calculated by the ratio of the area under the curve (AUC) of the amylose branches to that of the whole SEC weight CLD [31].

### 4.7. Apparent Amylose and Protein Contents and Gel Consistency

The apparent amylose content was determined by the iodine reagent method [10], and gel consistency was determined by the method of Cagampang et al. [32]. Protein fractions (albumin, globulin, prolamin and glutelin) were measured by the method of Ning et al. [33]. Total protein content was calculated by the sum of the four protein fraction contents. Three replicates were performed for each sample.

### 4.8. Statistical Analysis

The analysis of variance with Duncan’s multiple range test (*p* < 0.05) was performed using SPSS 17.0 software (Statistical Product and Service Solutions, IBM, NY, USA). Significant difference was evaluated based on *p* < 0.05.

## 5. Conclusions

The effects of the N level on the pasting properties, contents of the apparent amylose, protein and its fractions and starch structure, as well as the taste parameters of two indica-japonica hybrid rice cultivars differing in eating quality, were investigated in this study. The results showed that the appropriate nitrogen fertilizer (100 N kg/ha) did not worsen the rice-eating quality. Excessive N fertilizer dramatically increased the ratio of IG, amylose chains and water-insoluble proteins, especially prolamin, which is a major factor resulting in the deterioration of the eating quality of the two cultivars. Similarly, the inferior eating quality of IG was mainly attributed to high globulin and prolamin contents. The response of the eating quality to the N level was more sensitive for YY17 than YY12.

## Figures and Tables

**Table 1:** Rapid Visco Analyser (RVA) characteristics and taste traits under different nitrogen levels.

Cultivar	Treatment	RVA Characteristics (cP)							Taste Traits					GC (mm)
		PV	HPV	CPV	BD	SB	GT (°C)	PT (min)	Appearance	Hardness	Viscosity	Balance	Taste	
YY12	N0	3502cd	1605a	2924a	1897c	−578bc	76.3bc	5.91a	5.70c	6.88f	6.12b	5.83c	65.08c	55.3d
	LN	3434d	1524bcd	2832bc	1910c	−602c	76.7abc	5.84a	5.23d	7.12e	5.67c	5.37d	62.07d	48.2e
	MN	3302e	1505cd	2782cd	1797d	−520ab	76.6abc	5.87a	4.38g	7.52c	4.82e	4.45f	56.58f	46.8e
	HN	3225f	1471d	2731d	1754d	−494a	76.1c	5.82a	3.53h	7.92a	3.97g	3.57h	51.07h	44.0f
	Average	3366	1527	2817	1839	−548	76.4	5.86	4.71	7.36	5.14	4.8	58.7	48.6
YY17	N0	3769a	1595ab	2893ab	2174a	−876e	76.6abc	5.76a	7.22a	6.23g	7.57a	7.32a	74.47a	77.5b
	LN	3738a	1578ab	2817bc	2160a	−921e	76.9ab	5.76a	5.97b	6.78f	6.32b	6.07b	66.55b	84.8a
	MN	3639b	1630a	2857abc	2009b	−782d	76.9ab	5.82a	4.98f	7.23d	5.20d	5.02e	59.93e	68.2c
	HN	3551c	1569abc	2801cd	1982b	−751d	77.1a	5.76a	4.03g	7.65b	4.23f	4.02g	53.82g	57.0d
	Average	3674	1593	2841	2081	−832	76.89	5.77	5.55	6.98	5.83	5.6	63.69	71.9

YY12, Yongyou12; YY17, Yongyou17; N0, control; LN, low nitrogen; MN, medium nitrogen; HN, high nitrogen; PV, peak viscosity; HPV, hot paste viscosity; CPV, cool paste viscosity; BD, breakdown; SB, setback; GT, gelatinization temperature; PT, peak time and GC, gel consistency. Values with different letters in a column represent a significant difference (*p* < 0.05).

**Table 2:** Apparent amylose content and four protein contents under different nitrogen levels (g/100 g flour, %).

Cultivar	Treatment	AAC	Albumin	Globulin	Prolamin	Glutelin	Total Protein	Pro/Pro+Glu
YY12	N0	14.04a	0.28b	0.27d	0.57cd	3.96e	5.08e	0.127a
	LN	13.52ab	0.29ab	0.31bc	0.61c	4.26d	5.47d	0.125a
	MN	13.54ab	0.29ab	0.32b	0.65b	4.63c	5.89c	0.123ab
	HN	12.28bc	0.31a	0.37a	0.69a	4.93b	6.30b	0.123ab
	Average	13.34	0.29	0.32	0.63	4.44	5.69	0.13
YY17	N0	13.34ab	0.28b	0.29cd	0.52e	4.03de	5.11e	0.114cd
	LN	12.83abc	0.27b	0.29cd	0.55de	4.17de	5.28de	0.116bc
	MN	11.43c	0.28b	0.32b	0.60c	4.62c	5.81c	0.115cd
	HN	11.19c	0.31a	0.38a	0.65b	5.46a	6.80a	0.106d
	Average	12.20	0.28	0.32	0.58	4.57	5.70	0.114

AAC, apparent amylose content and pro/pro+glu, the ratio of prolamin to prolamin+glutelin. Values with different letters in a column represent a significant difference (*p* < 0.05).

**Table 3:** Starch molecular parameters extracted from size exclusion chromatography.

Cultivar	Treatment	AC	X_AP1_	X_AP2_	X_AM1_	X_AM2_	h_AP2/AP1_	h_AM1/AP1_	h_AM2/AP1_
YY12	N0	20.89abc	14.1a	38.9abc	ud	787.8bc	0.475bc	ud	0.127ab
	LN	21.57ab	14.1a	38.9abc	248.4ab	862.0abc	0.481abc	0.080abc	0.129ab
	MN	20.74bcd	14.0a	38.9abc	254.1a	904.2a	0.472c	0.076abcd	0.122bc
	HN	22.79a	14.0a	38.6c	237.9abc	923.3a	0.473c	0.085ab	0.136a
	Average	21.5	14.1	38.9	246.8	869.332	0.475	0.081	0.128
YY17	N0	18.52d	14.0a	39.0ab	233.9abc	868.2ab	0.484ab	0.064d	0.109d
	LN	18.58d	14.0a	39.2a	237.9abc	773.9c	0.488a	0.072bcd	0.107d
	MN	19.25cd	14.2a	39.1ab	218.8c	781.4bc	0.486a	0.070bc	0.112cd
	HN	21.39ab	14.1a	38.8bc	228.3bc	843.2abc	0.488a	0.087a	0.121bc
	Average	19.43	14.1	39.1	229.7	816.7	0.487	0.073	0.112

ud, undetectable; AC, amylose content; X_AP1_, X_AP2_, X_AM1_ and X_AM2_ stand for the degree of polymerization (DP) at peaks AP1, AP2, AM1 and AM2, respectively; h_AP2/AP1_, h_AM1/AP1_ and h_AM2/AP1_ stand for the ratio of peaks AP2, AM1 and AM2 height to peak AP1 height, respectively. Values with different letters in a column represent a significant difference (*p* < 0.05).

**Table 4:** Characteristics of superior and inferior grains under different nitrogen levels.

Cultivar	Treatment	Ratio of Whole Panicle (%)	Grain Length (mm)	Grain Width (mm)	Grain Thickness (mm)	Length/Width	Grain Weight (g)	GRK (%)	DRK (%)	NRK (%)
YY12	N0-SG	23.05c	5.86a	2.80a	1.83b	2.09d	22.38a	ud	ud	100.00a
	LN-SG	20.88d	5.84a	2.78ab	1.83b	2.10cd	21.72b	ud	ud	100.00a
	MN-SG	20.70d	5.69b	2.78ab	1.85a	2.04e	21.53b	0.17e	ud	99.83a
	HN-SG	21.26d	5.65b	2.77b	1.85a	2.04e	21.24b	0.67e	ud	99.33a
	Average	21.47	5.76	2.79	1.84	2.07	21.72	0.21	ud	99.79
	N0-IG	42.10b	5.47c	2.57c	1.62d	2.13bc	18.01c	24.17d	5.83d	70.00b
	LN-IG	44.14a	5.49c	2.53d	1.65c	2.17a	17.60c	28.83c	10.83c	60.33c
	MN-IG	45.93a	5.49c	2.54cd	1.62d	2.16ab	17.10c	36.17b	14.00b	49.83d
	HN-IG	44.05a	5.32d	2.52d	1.55e	2.11cd	16.74d	42.17a	16.00a	41.83e
	Average	44.06	5.44	2.54	1.61	2.14	17.36	32.83	11.71	55.46
YY17	N0-SG	21.09d	6.26a	2.65a	1.81a	2.36b	23.05a	ud	ud	100.00a
	LN-SG	17.76e	6.26a	2.65a	1.80b	2.36b	22.54b	ud	ud	100.00a
	MN-SG	17.64e	6.24a	2.65a	1.81a	2.35b	22.33b	ud	0.17e	99.83a
	HN-SG	16.00e	6.15b	2.61b	1.81a	2.35b	22.34b	ud	0.17e	99.83a
	Average	18.12	6.23	2.64	1.81	2.36	22.56	ud	0.08	99.92
	N0-IG	42.91c	5.92c	2.47c	1.69c	2.39a	19.71b	10.00c	9.17c	80.83b
	LN-IG	44.49c	5.85d	2.47c	1.66d	2.37ab	19.03c	17.17b	6.33d	76.50c
	MN-IG	46.73b	5.94c	2.49c	1.66d	2.38ab	18.50d	16.33b	11.00b	72.67d
	HN-IG	51.38a	5.84d	2.46c	1.63e	2.38ab	18.13e	41.83a	12.33a	45.83e
	Average	46.38	5.89	2.46	1.66	2.38	18.84	21.33	9.71	68.96

SG, superior grains; IG, inferior grains; GRK, green rice kernel; DRK, died rice kernel and NRK, normal rice kernel. Values in the same column followed by different letters in the same cultivar are significantly different (*p* < 0.05).

## Supplementary Materials

The following are available online at https://www.mdpi.com/2223-7747/9/12/1663/s1, Figure S1: The hot paste viscosity (**A**), cool paste viscosity (**B**), appearance (**C**), hardness (**D**), balance (**E**), total protein content of superior and inferior grains under different nitrogen levels. YY12, Yongyou12; YY17, Yongyou17; SG, superior grains; IG, inferior grains; N0, control; LN, low nitrogen; MN, medium nitrogen; HN, high nitrogen; Different letters labeled on the columns in the same cultivar are significantly different (*p* < 0.05); Figure S2: Comparison of brown rice appearance under different N levels. NRK, Normal rice kernel (in red rectangle); GRK, green rice kernel (in blue rectangle); DRK, died rice kernel, (in orange rectangle).
