# Supplementary material for: The Influence of Nitrogen Application Level on Eating Quality of the Two Indica-Japonica Hybrid Rice Cultivars
Source: Plants (Basel). 2020 Nov 27;9(12):1663. doi: 10.3390/plants9121663 (PMC7760565; doi:10.3390/plants9121663)
Supplement: Supplementary file 1 [file plants-09-01663-s001.pdf]

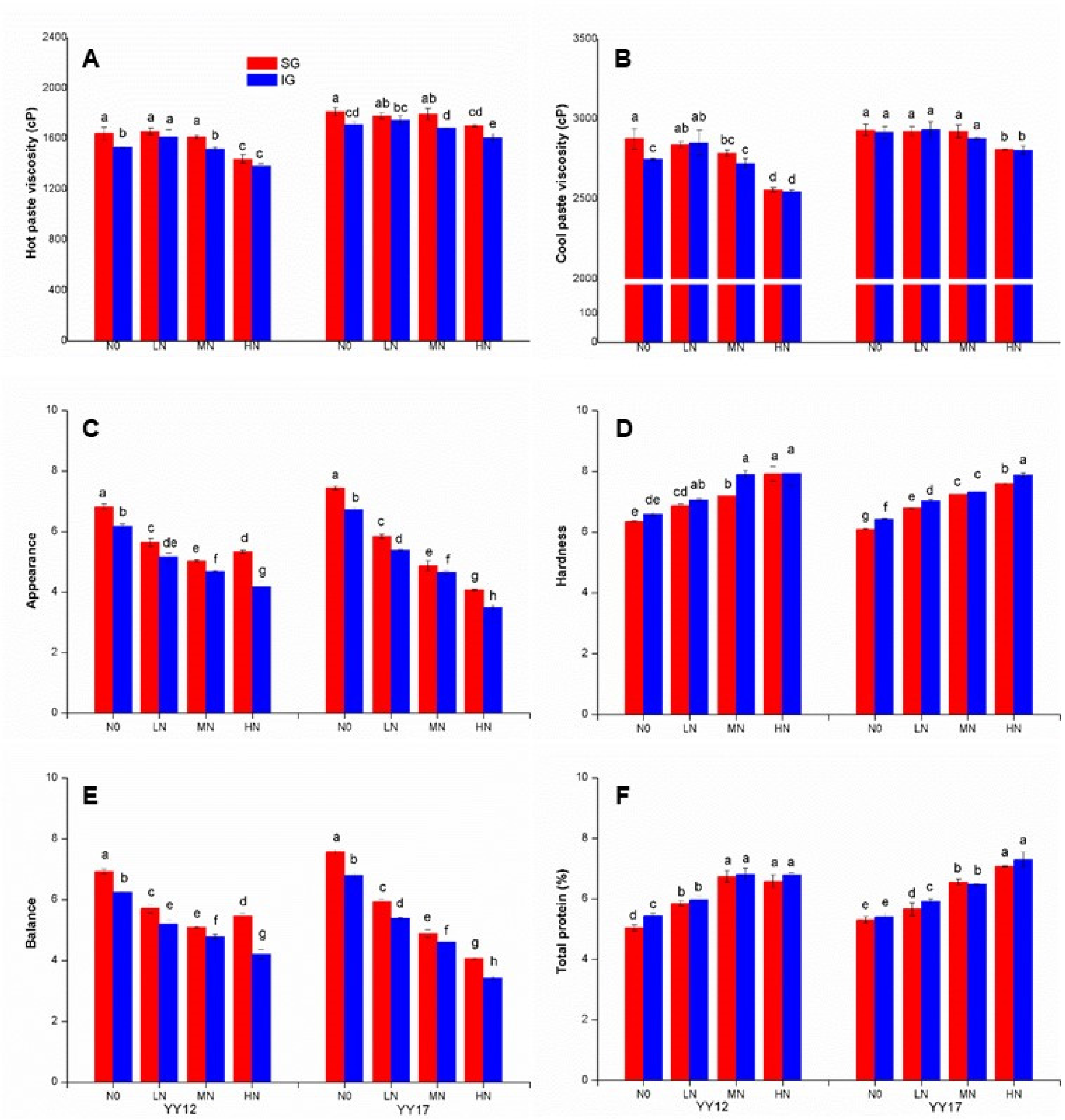

Figure. S1. The hot paste viscosity (**A**), cool paste viscosity (**B**), appearance (**C**), hardness (**D**), balance (**E**), total protein content of superior and inferior grains under different nitrogen levels. YY12, Yongyou12; YY17, Yongyou17; SG, superior grains; IG, inferior grains; N0, control; LN, low nitrogen; MN, medium nitrogen; HN, high nitrogen; Different letters labeled on the columns in the same cultivar are significantly different ($p < 0.05$).

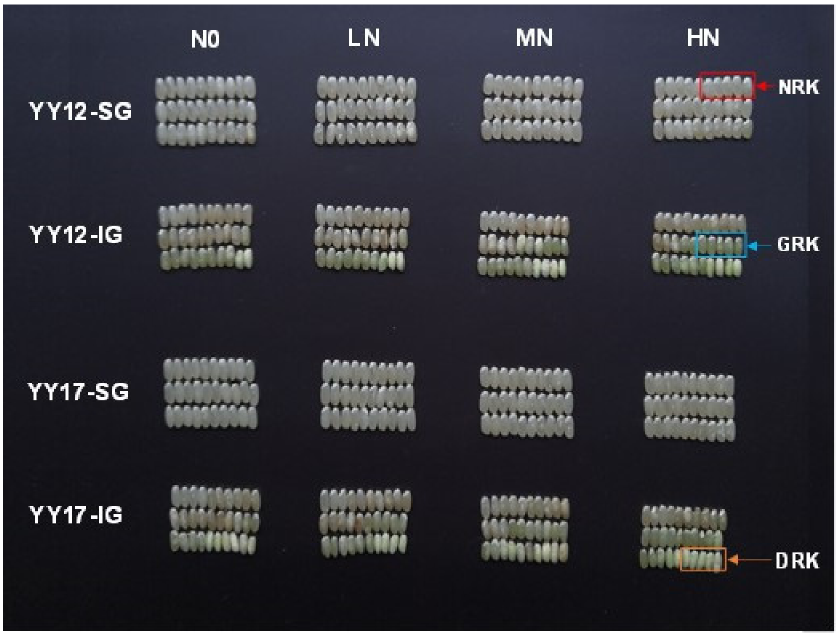

Figure. S2. Comparison of brown rice appearance under different N levels. NRK, Normal rice kernel (in red rectangle); GRK, green rice kernel (in blue rectangle); DRK, died rice kernel, (in orange rectangle).
